# Pituitary incidentalomas - How often is too often?

**Published:** 2009

**Authors:** Dumitru Ferechide, Daniela Radulescu

**Affiliations:** * “Carol Davila” UMP, Department of Physiology, Bucharest; ** “Carol Davila” UMP, “Sf. Ioan” Clinical Hospital, Bucharest

**Keywords:** CRF (chronic renal failure), Hyperhomocysteinemia, Cardiovascular disease

## Abstract

Homocysteine is a sulfurated amino acid used for the synthesis of methionine. The last decade’s researches proved that hyperhomocysteinemia is an independent risk factor for atherosclerotic vascular disease. The vascular injury induced by several mechanisms of hyperhomocysteinemia is the hallmark of homocysteine’s atherogenic properties. Hyperhomocysteinemia is present in 85% of the patients with chronic renal failure (cardiovascular diseases are the main cause of mortality) and persists after initiating dialysis or after renal transplantation. Although folic therapy or folinic acid therapy reduce homocysteine levels with 20-40% in hemodialysis patients, the effects on cardiovascular morbidity have yet to be proven in future studies.

Cardiovascular diseases represent the first cause of death in patients with chronic renal failure. Taking this into consideration, the last decade researches revealed supplementary explanations against the classical ones. Nowadays, it has been proven that hyperhomocysteinemia is a risk factor for atherosclerosis and cardiovascular complications independent of other factors, both in patients with chronic renal failure (CRF) and in non renal ones. 

## Homocysteine metabolism

Homocysteine is a sulfurated amino acid used intracellularily for the methionine synthesis within the so-called methionine-homocysteine cycle. The methionine is used for the intracellular protein synthesis or it can be transformed into S-adenosylmethionine under the enzymatic action of adenosyle methionine transferase, ATP-consuming reaction. S-adenosylmethionine is used as a methyl groups donor in transmethylation reactions; after demethylation, the result being the adenosylhomocysteine thioether, which reacts as a potent inhibitor of all demethylation reactions of S-adenosylmethionine and which is – reversibly - hydrolyzed in homocysteine and adenosine. 

Homocysteine catabolization may follow two pathways (**Table 1**):

- The transsulfuration pathway takes place under the action of cystathion-β-synthetase which binds the serine with homocysteine and leads to the formation of cystathionine; the cystathionine is cleaved, under the action of a cystationase dependent on B6 vitamin, into 2-ketobutirate and cysteine. The cysteine is subsequently converted into glutathione, taurine and other sufurated amino acids.

- The remethylation of homocysteine conducts to the synthesis of methionine, which takes place under the action of methyltetrahydrofolat-homocysteine methyltransferase (methionine-synthetase), having as co-factors the active folats and B12 vitamin; methyl grouping results either from methyltetrahydrofolat (the reduction outcome of the methyltetrahydrofolat, under the action of methylenetetrahydrofolate reductase) or from betaine.

The reaction of transsulfuration predominates in the states of satiety, while the one of remethylation predominates in fasting states. S-adenosylmethionine is the crucial factor which determines the predomination of one of the two reactions of homocysteine metabolization, through the inhibition of methylenetetrahydrofolate reductase and the activation of cystathion-β-synthetase. 

Homocysteine is found in the plasma in oxidized form in a proportion of 98% and under reduced form in 2%; the most part of the oxidized form circulates bind to proteins, the rest being free.

The sum of all the forms – oxidized bound to proteins, free reduced oxidized – affects the total homocysteinemy. The normal levels of plasmatic homocysteine are between 5-15μmol/L.

Hyperhomocysteinemia can be classified in mild (16-30μmol/L), moderate (31-100μmol/L) and severe (over 100μmol/L).

In order to avoid the false positive reactions, homocysteine dosage will be made from plasma immediately after the sampling and centrifugation of the blood, due to the liberation of homocysteine from erytrocites in an amount of 5 μmol/hour.

The methionine test can be performed à jeun in persons with risk and normal homocysteinemy [**1**]: 100mg/kg methionine is administered, then the plasma homocysteine levels are measured after 4, 8 hours respectively; the normal levels are shown in **Table 1**. The test is considered positive if, after 4 hours, homocysteinemia exceeds the value considered to be normal with at least two standard deviations. The methionine test is important especially in patients with cystathion-β-synthetase deficiency and has less value in those with methylenetetrahydrofolate reductase deficiency.

**Table 1:** The methionine stimulation test

Sex	Basic Values (μmol/L)	At 4 hours (μmol/L)	At 8 hours (μmol/L)
Men	6.2	16.3	17.5
Women			
­- premenopause	6.3	14.7	17.6
­- postmenopause	4.8	22.4	24.7

## Causes of hyperhomocysteinemy 

1. Genetic causes:

- In homocystinuria (the deficiency of cystathion-β-synthetase), homocysteinemia à jeun exceeds the normal values 40 times and is associated with hypermethioninemia. Clinical features include: delay in growth, osteoporosis, ocular anomalies, severe and premature atherosclerosis, thromboembolic complications; death occurs at young ages, mainly due to cardiovascular causes. The treatment consists of large doses of B6 vitamin which activate the minimum amount of available enzyme.

- The deficiency of methionine synthetase

- The deficiency of methylenetetrahydrofolate reductase (MTHFR)

- Mutations of MTHFR gene may be accompanied by hyperhomocysteinemia when associated with reduced dietary folats intake.

2. Alimentary causes: deficiency of folats, B6 vitamin and/or B12 vitamin through:

- decreased intake (vegetarians, prolonged boil of food) 

- deficit of absorption (alcoholism, gastric atrophy) 

- increased requirements (children)

3. Medicinal:

- drugs which inhibit dihydrofolate reductase (methotrexate, trimetoprim) 

- cholestyramine and colestipole reduce the absorption of folats and cobalamin

- fenitoine and carbamazepine are antagonists with folats

- niacine, teofiline reduce kinases dependent on B6 vitamin 

4. Chronic hepatic disease

5. CRF is associated in 85% of the hyperhomocysteinemy cases 

**Fig. 1:**
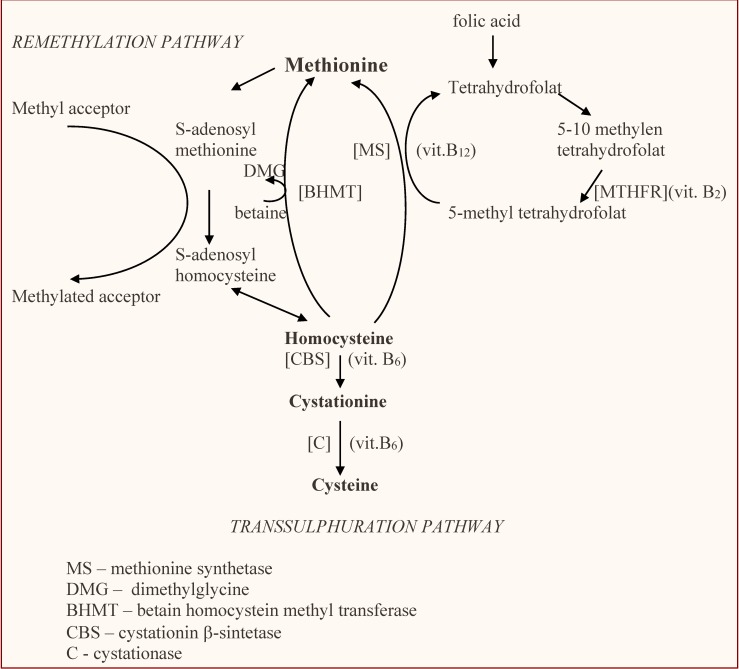
Homocysteine metabolism

## Hyperhomocysteinemy as cardiovascular risk factor

The connection between hyperhomocysteinemy and atherosclerotic coronary, peripheral or cerebral vascular diseases has been demonstrated in numerous studies.

A meta-analysis of 31 studies, comprising approximately 7,000 patients, emphasized that the serum levels of homocysteine are increased with 30-90% in patients with cerebral, peripheral or coronary vascular diseases as compared to the control groups. This meta-analysis has also determined that the risk of ischemic coronary disease in patients with hyperhomocysteinemy is 30.8 higher than in patients with normal homocysteinemy [**2**].

Homocysteine serum levels are correlated with the risk of myocardial infarction. In a study on 14,916 male physicians with no infarction in the last 5 years before the study (Physicians Health Study 1992), the risk of myocardial infarction in patients with hyperhomocysteinemy was 3 times higher as compared to the ones with normal homocysteinemy; in addition, the authors estimated that approximately 7% of the infarctions that happened during the study were due to hyperhomocysteinemy as single cardiovascular risk factor [**3**].

In Framingham’s study, the risk of extra-cranial carotid stenosis (over 25% of the lumen) was significantly increased in patients with homocysteinemy comprised between 11.4-14.3 μmol/L, values, previously considered as being normal [**4**].

Hyperhomocysteinemy has proved to have a direct relation with the global mortality, especially in type 2 diabetes. In a study [**5**] on 2,484 persons aged between 50-75 years old, the risk of death with homocysteinemy over 14 μmol/L was 1.56 times higher than in patients with normal homocysteinemy. The relation was more evident in type 2 diabetes as the risk of death was 2.51 times higher compared to 1.34 in nondiabetics with homocysteinemy over the mentioned value. 

**Fig. 2:**
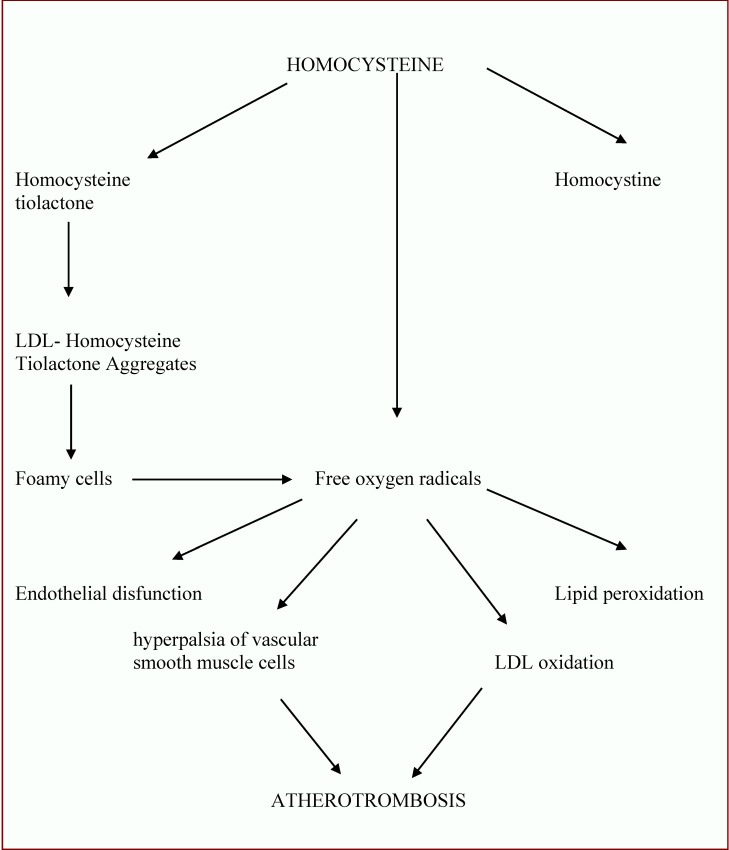
The hypothetical mechanism of the hemocysteine-induced atherogenesis [**10**]

The mechanism through which hyperhomocysteinemy stimulates the atherogenesis and has trombogenic properties is incompletely known. The vascular injury induced by hyperhomocysteinemy seems to be the central element of the atherogenesis induced by this; the mechanisms of vascular injury are multiple (**Figure 2**):

- thyolactone metabolite of homocysteine binds with LDL cholesterol and the formation of aggregates which are taken over by the vascular macrophages in the arterial intima. The result - foamy cells which release lipids in the atherom plaque [**6**,**7**].

- the prolonged exposure of endothelial cells to homocysteine lowers the secretion of nitric oxid with secondary alteration of vasodilatation [**8**].

- free oxygen radicals, formed during the oxidation of the reduced homocysteine, have a direct toxic effect on the vascular endothelial cells [**9**]. 

- the increase of the blood viscosity and of the medium arterial blood pressure as a response to acute hyperhomocysteinemy [**9**].

- homocysteine has protrombotic effects by activating factor V: the inhibition of proteine C and of the heparan-sulfate, the increase of the fibrinopeptid A and of the fragments 1 and 2 of prothrombin, the decrease of the antitrombotic activity of endothelium through the alteration of thrombomodulin function [**9**].

- accumulation of trombocites secondary to the direct proaggregant effects of homocysteine or secondary to the alteration of hemocysteine – induced vasodilatation. 

- hyperhomocysteinemy can activate the cascade of coagulation in patients with acute coronary syndromes by increasing factor VIIa and by generating thrombin [**10**].

Most of the experimental studies which have demonstrated that those harmfull effects refer to important increases of homocysteine (between 100-1000 μmol/L) by reduced fraction, which is not true in renal patients, were the oxidized fraction increases. Some authors consider the increase of the oxidized fraction of plasmatic homocysteine only a marker of the metabolic anomalies of amino acids due to CRF and characterize it as being an “innocent bystander”. The way hyperhomocysteinemy increases the risk of cardiovascular diseases in CRF is still unknown. There are studies which demonstrate that the toxic effect of moderate hyperhomocysteinemy on endothelial cells appears only when these previous injuries have other causes (as in CRF or in dialysed patients) or when these have increased susceptibility to injuries (as in heterozygotes with cystathion-β-synthetase deficiency). 

## Hyperhomocysteinemy in crf

Moderate hyperhomocysteinemy is noted from the early stages of CRF, becoming more prominent as the renal function deteriorates; hyperhomocysteinemy persists after the initiation of hemodialysis or peritoneal dialysis or after the renal transplantation [**11**]. The increase exceeding the normal levels of serum homocysteine is noticed not only in adults but also in children with CRF [**11**].

The increase of homocysteine is done by oxidized fraction, the reduced one being normal or even low. 

75-90% of the chronic hemodialysed patients have hyperhomocysteinemy [**12**]; each 1 μM increase of serum homocysteine is proven to associate with an increase of 4% in thrombosis risk of the arteriovenous fistula [**13**]; the risk of myocardial infarction is 5 to 10 times higher in patients with CRF than in general population. As a result, the therapeutic reduction of homocysteine serum level would bring a notable benefit. 

The causes of hyperhomocysteinemy in chronic dialysed patients may be multiple: the decreased renal clearance (the alteration, especially, of the tubular function), the alteration of homocysteine metabolism (the increase of the peroxidation processes), undiagnosed genetic anomalies (cystathion-β-synthetase deficiency or MTHFR deficiency) and/or vitamins deficiency (B6, B12 or folic acid). Persistence of hyperhomocysteinemy after transplantation, even in patients with good renal function, is not yet explained; some authors incriminated the therapy with cyclosporine A of being an additional factor of hyperhomocysteinemy [**14**] but this hypothesis has not been confirmed by all the studies [**15**].

In the last 10 years there has been demonstrated the possibility of reducing the homocysteine levels with 20-40% in hemodialysed patients after the administration of folic acid 1-15 mg/day, even in patients without folats deficiency. The capacity of folic acid to reduce the serum homocysteine is due to the stimulation of homocysteine remethylation in methionine. The efficiency of folic acid is limited, the normalization of homocysteinemy being obtained only on an insignificant proportion of the dialysed patients, even with maximal doses and by obtaining a folats serum level of over 20-50 times than normal. These observations raised the hypotesis that dialysed patients present a resistance to the folic acid therapy, probably due to the disorder of folic acid metabolization at all levels: the reduction of deconjugation in the intestinal cells by inhibiting the activity of conjugases (γ gluthamyl carboxypeptidase) in the uremic environment, the reduction of intestinal absorption of methyltetrahydrofolat, the inhibition of transmembranar transport by the uremic toxins. This hypothesis is sustained by researches which have demonstrated that oral or parenteral therapy with folinic acid – the reduced form of folat (methyltetrahydrofolat) – has an efficiency that is superior to folic acid [**16**]. Hemofiltration or daily nocturnal hemodialysis were proven to be superior to conventional hemodialysis, in reducing hyperhomocysteinemy [**17**]. Hyperhomocysteinemy is also present in peritoneal dialysed patients; although homocysteine is removed by peritoneal dialysis, the amount is innappropiate and the treatment with folic acid is necessary [**18**]. Nowadays, the administration of folic acid in chronic dialysed patients has become a routine practice, but the conclusion of the large randomized studies is inconsequent as far as the effect on the cardiovascular mortality and morbidity rates are concerned [**17**].

The homocysteine decrease of folic acid after therapy is smaller in patients with vascular disease than in non-vasculars (after a three months’ treatment of 1-5 mg/day folic acid, a decrease of 34% in patients with no vascular disease and only 2.5% in those with vascular disease for over 10 years; the normalization of homocysteine was obtained in 27% in the first group against 6% in the second one [**19**]), irrespective of age, previous deficit of folats, B6, B12 or other factors. In 1996 [**20**], Robinson and collab. published a study in which homocysteine serum levels were higher in uremics with cardiovascular complications (29.9 ± 1.3 μmol/L) than in uremics without vascular diseases (23.9 ± 1.5 μmol/L).

The difference between the responses to folic acid therapy in patients with CRF and vascular disease raises three hypotheses:

- is homocysteine metabolism modified in these patients?

- is hyperhomocysteinemy a marker of an alternative metabolization which predisposes to cardiovascular disease?

- is hyperhomocysteinemy itself a marker of cardiovascular disease?

Those hypotheses were also raised by other studies which came to the conclusion that the connection between homocysteinemy and cardiovascular diseases depend on the complex interaction between the environmental factors, genetic factors and nutritional factors. Recent studies have demonstrated that the genetic mutations of some enzymes involved in homocysteine metabolization, as methylenetetrahydrofolate reductase (MTHFR) or cystathion-β-synthetase (CBS), they can modulate the response of homocysteine reduction after the administration of folic acid and can predispose to cardiovascular complications. Kruger and collab. have emphasized that certain elements of CBS are associated with a decreased response to folic acid treatment and an increased risk of developing cardiovascular diseases. However, the increase of folic acid partly corrects the lack of response concerning the decrease of homocysteinemy, which will plead for a polymorphism of CBS genes [**21**].
